# Microbial Community Composition in Municipal Wastewater Treatment Bioreactors Follows a Distance Decay Pattern Primarily Controlled by Environmental Heterogeneity

**DOI:** 10.1128/mSphere.00648-21

**Published:** 2021-10-20

**Authors:** Taegyu Kim, Sebastian Behrens, Timothy M. LaPara

**Affiliations:** a Department of Civil, Environmental, and Geo-Engineering, University of Minnesota Twin Cities, Minneapolis, Minnesota, USA; b Biotechnology Institute, University of Minnesota Twin Cities, St. Paul, Minnesota, USA; University of Michigan—Ann Arbor

**Keywords:** wastewater treatment, activated sludge, distance decay, microbial biogeography pattern, microbiome

## Abstract

Understanding spatiotemporal patterns in microbial community composition is a central goal of microbial ecology. The objective of this study was to better understand the biogeography of activated sludge microbial communities, which are important for the protection of surface water quality. Monthly samples were collected from 20 facilities (25 bioreactors) within 442 km of each other for 1 year. Microbial community composition was characterized by sequencing of PCR-amplified 16S rRNA gene fragments. Statistically significant distance decay of community similarity was observed in these bioreactors independent of clustering method (operational taxonomic units [OTUs] at 97% similarity, genus-level phylotypes) and community dissimilarity metric (Sørensen, Bray-Curtis, and weighted Unifrac). Universal colonizers (i.e., detected in all samples) and ubiquitous genus-level phylotypes (i.e., detected in every facility at least once) also exhibited a significant distance decay relationship. Variation partitioning analysis of community composition showed that environmental characteristics (temperature, influent characteristics, etc.) explained more of the variance in community composition than geographic distance did, suggesting that environmental heterogeneity is more important than dispersal limitation as a mechanism for determining microbial community composition. Distance decay relationships also became stronger with increasing distance between facilities. Seasonal variation in community composition was also observed from selected bioreactors, but there was no clear seasonal pattern in the distance decay relationships.

**IMPORTANCE** Understanding the spatiotemporal patterns of biodiversity is a central goal of ecology. The distance decay of community similarity is one of the spatial scaling patterns observed in many forms of life, including plants, animals, and microbial communities. Municipal wastewater treatment relies on microorganisms to prevent the release of excessive quantities of nutrients and other pollutants, but relatively few studies have explored distance decay relationships in wastewater treatment bioreactors. Our results demonstrate a strong distance decay pattern in wastewater treatment bioreactors, regardless of the sequence clustering method or the community dissimilarity metric. Our results suggest that microbial communities in wastewater treatment bioreactors are not randomly assembled but rather exhibit a statistically significant spatial pattern.

## INTRODUCTION

Distance decay relationships are well-known, fundamental spatial patterns of biodiversity and have been recognized by ecologists for several decades (1–3). The bulk of our knowledge on distance decay relationships is based on plants (2, 4) and animals (1, 4), but the spatial distribution of microbes has become of interest. Recent empirical analyses of some patterns for microbial communities suggest that there are biodiversity scaling rules common to all forms of life (5); in fact, recent studies indicate that microbes also have a substantial distance decay pattern (5–12).

Two different mechanisms are used to explain the distance decay of community similarity: environmental heterogeneity and dispersal limitation (2, 13). As geographic distance increases, environmental conditions tend to be more different but also the dispersal limitation becomes stronger. Previous studies have attempted to determine which of these two interconnected variables is the main driver of microbial community distance decay patterns (environmental heterogeneity versus geographic distance) using experimentally manipulated enrichment cultures (14) and variation partitioning statistical analyses of natural ecosystems (4, 15). These studies showed that the distance decay of microbial community composition was caused more by environmental heterogeneity than by limited dispersal.

Wastewater treatment bioreactors support one of the most complex microbial ecosystems ever applied for a specific purpose (16). Many efforts have been made to understand the microbial communities in these bioreactors, and understanding their spatial patterning and underlying mechanisms controlling microbial community composition is emerging. Recently, a weak distance decay relationship was observed with microbial communities from 26 wastewater treatment bioreactors (as far as ∼3,000 km between two bioreactors) (17); another study observed a statistically significant distance decay relationship in wastewater bioreactor communities at a global scale (18).

The objective of this study was to characterize the distance decay relationships among the complex bacterial communities growing in municipal wastewater treatment bioreactors at relatively short distances (<500 km). Because prior research has demonstrated that bioreactor community assembly is largely deterministic (19, 20), we hypothesize that wastewater treatment bioreactors would exhibit a strong distance decay relationship, particularly at shorter distances than has been previously studied. To test this hypothesis, samples from 20 full-scale wastewater treatment facilities from within the state of Minnesota were collected each month for a year. Microbial community composition was determined by sequencing of PCR-amplified 16S rRNA gene fragments. Community dissimilarity, richness, and specific genus-level phylotype abundances were then studied along with bioreactor characteristics and geographic locations. The spatial community shift rate (β) was calculated for the whole community and the *Archaea*. The β was also calculated only considering universal and ubiquitous genus-level phylotypes to ascertain distance decay relationships excluding the effects of limited dispersal (i.e., if these microbes were detected at all locations, then dispersion was not a pertinent factor).

## RESULTS

### Community richness and dissimilarity.

Samples were collected monthly from 20 different wastewater treatment facilities (25 bioreactors) for 1 year. Annual average operating parameters and facility characteristics are summarized in Tables S1 and S2 in the supplemental material, respectively. Distances between the facilities ranged from 11 km to 442 km; the relative distances among the facilities are depicted in Fig. S1 in the supplemental material. A total of 292 activated sludge samples were collected, and their extracted DNA was used as the template for sequence analysis of PCR-amplified 16S rRNA gene fragments. An average of 24,419 (standard deviation [SD] = 9,626) quality sequences were obtained per sample. Three samples that had less than 4,336 quality sequences were discarded prior to operational taxonomic unit (OTU)-based analysis; 8 samples that had less than 7,805 quality sequences were discarded prior to analysis of genus-level phylotypes. This analysis generated an average of 2,030 OTUs per sample (SD = 302) and 262 genus-level phylotypes per sample (SD = 25).

10.1128/mSphere.00648-21.1 TABLE S1 Annual average operational parameters. Unmarked units are milligrams per liter. Abbreviations: OTU, operational taxonomic unit; CBOD, carbonaceous biochemical oxygen demand; TSS, total suspended solids; TKN, total Kjeldahl nitrogen. Download Table S1, DOCX file, 0.1 MB. Copyright © 2021 Kim et al. 2021 Kim et al. https://creativecommons.org/licenses/by/4.0/ This content is distributed under the terms of the Creative Commons Attribution 4.0 International license.

10.1128/mSphere.00648-21.2 TABLE S2 Facility characteristics and design components. Download Table S2, DOCX file, 0.1 MB. Copyright © 2021 Kim et al. 2021 Kim et al. https://creativecommons.org/licenses/by/4.0/ This content is distributed under the terms of the Creative Commons Attribution 4.0 International license.

10.1128/mSphere.00648-21.6 FIG S1 Approximate relative geographic distances between 20 facilities, plotted using longitude and latitude. Distances between the facilities ranged from 11 km to 442 km. Download FIG S1, DOCX file, 0.04 MB. Copyright © 2021 Kim et al. 2021 Kim et al. https://creativecommons.org/licenses/by/4.0/ This content is distributed under the terms of the Creative Commons Attribution 4.0 International license.

**FIG 1:**
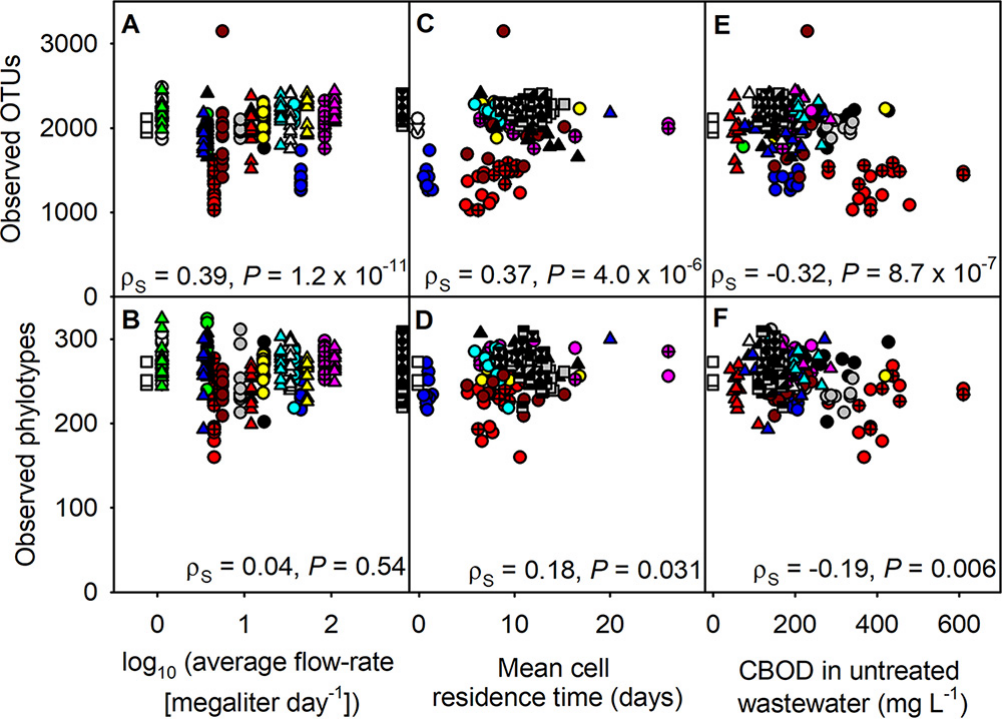
The number of observed operational taxonomic units (OTUs) and genus-level phylotypes as a function of wastewater flow rate (A and B), mean cell residence time (C and D), and carbonaceous biochemical oxygen demand (CBOD) in the untreated wastewater (E and F). Spearman’s correlation coefficients (ρ*_s_*) and *P* values are shown in each plot.

The diversity of bacterial communities growing in these wastewater treatment bioreactors varied as a function of several key parameters (Table 1). The number of observed OTUs had a statistically significant (*P* = 1.2 × 10^−11^) positive correlation with the average flow rate (∼facility size), although the number of observed genus-level phylotypes did not correlate with the average flow rate (*P* = 0.54) (Fig. 1). A statistically significant taxon-volume relationship was shown when the number of observed OTUs and the facility size were correlated in log scale (*z* = 0.03); however, this relationship was not statistically significant when genus-level phylotype-based community profiles were considered (Fig. S2).

**TABLE 1:** Spearman’s correlation coefficients between environmental variables and bioreactor community alpha-diversity indices^*a*^

Parameter or type of water	Environmental variable^*b*^	No. of observed OTUs	OTU Shannon diversity index	No. of observed phylotypes	Phylotype Shannon diversity index
Community richness	No. of observed OTUs		0.97*	0.47*	0.25*
	No. of observed phylotypes	0.47*	0.47*		0.61*

Operational parameter	Avg flow rate	0.39*	0.34*	0.04	−0.14
	pH	−0.22	−0.19	−0.16	−0.03
	Mean cell residence time	0.37*	0.35*	0.18	0.03

Untreated wastewater	CBOD	−0.32*	−0.32*	−0.19	−0.11
	TSS	0.11	0.12	−0.11	−0.15
	Phosphorus	−0.09	−0.08	−0.02	−0.14
	TKN	−0.02	−0.06	0.00	−0.04

Treated wastewater	pH	0.09	0.15	0.19	0.08
	CBOD	0.05	0.01	0.10	0.22
	TSS	−0.07	−0.07	−0.07	−0.06
	Phosphorus	−0.07	−0.04	−0.01	0.08
	NH_3_	−0.19	−0.20	0.01	0.11
	NO_3_ + NO_2_	0.17	0.16	−0.11	−0.15
	TKN	−0.33	−0.35*	0.01	0.12

a An asterisk indicates a statistically significant correlation (*P < *0.001).

b OTU, operational taxonomic unit; CBOD, carbonaceous biochemical oxygen demand; TSS, total suspended solid; TKN, total Kjeldahl nitrogen.

10.1128/mSphere.00648-21.7 FIG S2 Average flow rate and the number of observed OTUs (operational taxonomic units) (A) and the number of observed phylotypes in log scale (B). The taxon-volume relationship exponents (*z*; the slope from linear regression), the correlation coefficients (Pearson’s ρ), and the *P* values are shown in each figure. Download FIG S2, DOCX file, 0.1 MB. Copyright © 2021 Kim et al. 2021 Kim et al. https://creativecommons.org/licenses/by/4.0/ This content is distributed under the terms of the Creative Commons Attribution 4.0 International license.

The number of observed OTUs also had a significant positive correlation with mean cell residence time and exhibited a significant negative correlation with carbonaceous biochemical oxygen demand (CBOD) in the untreated wastewater (Table 1). However, the correlation between the number of observed OTUs and these two variables became insignificant (*P* = 0.004 and 0.002, respectively) when partial correlations were performed with average flow rate as a control variable (Table S3). The average flow rate did not lose statistical significance in the partial correlation performed by controlling other variables. When correlations between the number of observed OTUs and these two environmental variables were made within each facility, no significant correlation was observed, possibly because of the low sample number (typically 11 or 12 samples were collected at each facility) and/or the variance of environmental characteristics was not substantial within each facility. Variance of the mean cell residence time and of the CBOD were generally larger between the facilities than within each facility. A visual inspection of the correlation between community richness and these two variables (Fig. 1) suggests that the correlations were driven by differences among the facilities, not by temporal variation within each facility.

10.1128/mSphere.00648-21.3 TABLE S3 Spearman’s partial correlation coefficients and *P* values between the number of observed OTUs and three environmental variables. An asterisk indicates statistically significant correlation (*P* < 0.001). CBOD, carbonaceous biochemical oxygen demand. Download Table S3, DOCX file, 0.04 MB. Copyright © 2021 Kim et al. 2021 Kim et al. https://creativecommons.org/licenses/by/4.0/ This content is distributed under the terms of the Creative Commons Attribution 4.0 International license.

The bioreactors studied here generally did not show seasonal cyclic patterns in alpha-diversity; monthly community alpha-diversity did not correlate with monthly average temperatures except for possibly one facility. Facility L showed seasonal cyclic changes in all four alpha-diversity measurements (i.e., observed number of OTUs, observed number of genus-level phylotypes, OTU-based Shannon diversity index, and Shannon diversity index using genus-level phylotypes) with strong correlations with monthly average temperature (Spearman’s rho [ρ*_s_*] = 0.66, 0.76, 0.78, and 0.81, respectively).

Bray-Curtis dissimilarity indices were computed using the community profiles of genus-level phylotypes (Fig. S3). Analysis of molecular variance (AMOVA) showed that bacterial community composition from different facilities had statistically different community compositions (all *P* < 0.001), which suggests that facility-to-facility variation in bacterial community composition is bigger than the temporal variance within an individual facility. In contrast, bacterial communities in parallel bioreactors within the same facility were not statistically distinguishable (all *P* > 0.001).

10.1128/mSphere.00648-21.8 FIG S3 Bray-Curtis dissimilarities between all the samples (phylotype-based community profile) visualized with principal coordinate analysis (PCoA). Download FIG S3, DOCX file, 0.1 MB. Copyright © 2021 Kim et al. 2021 Kim et al. https://creativecommons.org/licenses/by/4.0/ This content is distributed under the terms of the Creative Commons Attribution 4.0 International license.

### Distance decay of the whole community similarity.

**FIG 2:**
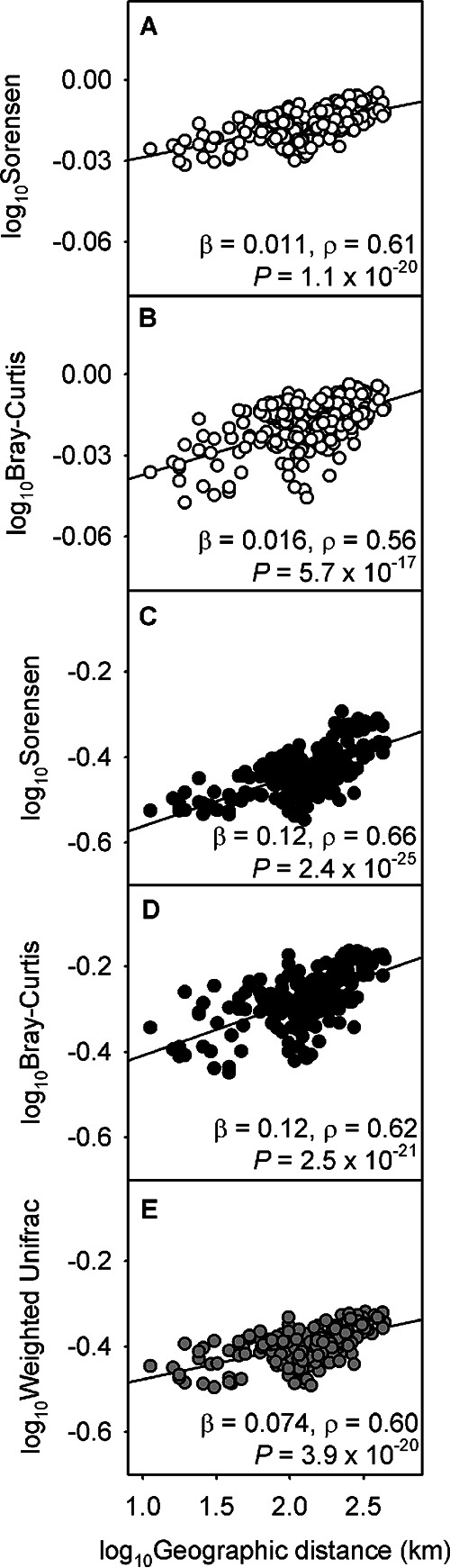
Log_10_-transformed distance and annual average operational taxonomic unit (OTU)-based community dissimilarities when calculated by using Sørensen index (A) and Bray-Curtis index (B). Log_10_-transformed distance and annual average community dissimilarities based on genus-level phylotypes when calculated by using Sørensen index (C) and Bray-Curtis index (D). (E) Log_10_-transformed distance and annual average weighted Unifrac distance. The slope of each linear regression determines the spatial community shift rate (β). The linearity and the statistical significance of each model were shown using the Pearson’s correlation coefficient (ρ) and *P* value, respectively.

There is a strong linear correlation between the log_10_-transformed annual average community dissimilarities (Sørensen, Bray-Curtis, and weighted Unifrac) and the log_10_-transformed geographic distances between paired facilities (Fig. 2). This suggests that community dissimilarity metrics based on presence/absence (Sørensen), relative abundance (Bray-Curtis), and phylogenetic distance (weighted Unifrac) exhibit distance decay relationships. The distance decay relationship calculated based on the OTU-based community profile had a lower β than the genus-level phylotype-based community profile by ∼10-fold, with both the Sørensen (0.011 versus 0.12) and Bray-Curtis (0.016 versus 0.12) metrics. The β calculated using phylogenetic distance (0.074) fell between the calculations based on OTUs and on genus-level phylotypes. Nonetheless, all sequence clustering methods exhibited statistically significant distance decay relationships (*P* < 10^−16^). Therefore, the qualitative conclusion (i.e., significant distance decay of community similarity) did not depend on either the dissimilarity metric or how the sequences were clustered.

### Distance decay of specific microbial groups.

Highly abundant members of the microbial communities were generally ubiquitous and persistent, whereas rare members were typically sparse and transient (Fig. S4). Universal colonizers were defined as genus-level phylotypes that were detected in all bioreactor samples analyzed in this study. There were 22 universal colonizers; the average relative abundance of universal colonizers was 37.9% (SD = 5.3%) of the total community in each sample. Ubiquitous phylotypes were defined as genus-level phylotypes that were detected in every facility on at least one occasion. There were 181 ubiquitous phylotypes, comprising an average of 72.3% (SD = 6.2%) of the microbial community in each sample. Finally, *Archaea* were examined in more detail; 34 different archaeal phylotypes were detected (arithmetic mean = 0.31%, SD = 0.23% in each sample as the relative abundance). Community dissimilarity matrices were constructed separately using each of these groups of organisms.

10.1128/mSphere.00648-21.9 FIG S4 (A) Number of bioreactors that were colonized by each phylotype (sorted by abundance rank) and (B) number of bioreactors that each phylotype was found. The correlation coefficients between phylotype abundance rank and number of bioreactors (Spearman’s ρ*_s_*) and the *P* values are shown in each figure. *y* = 25 at panel A were categorized as universal colonizer, and *y* = 25 at panel B were categorized ubiquitous phylotypes. Download FIG S4, DOCX file, 0.1 MB. Copyright © 2021 Kim et al. 2021 Kim et al. https://creativecommons.org/licenses/by/4.0/ This content is distributed under the terms of the Creative Commons Attribution 4.0 International license.

**FIG 3:**
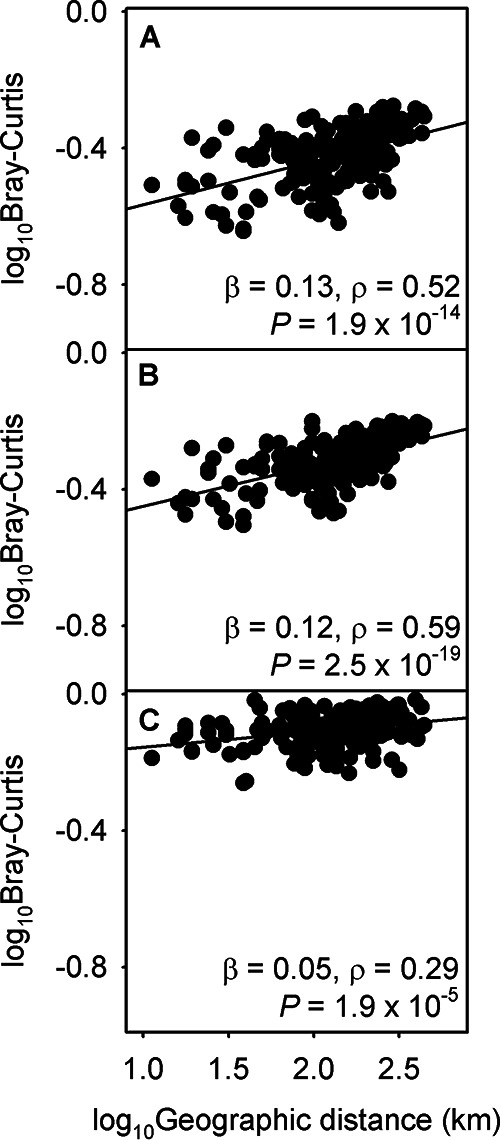
Log_10_-transformed distance and annual average dissimilarities calculated using the Bray-Curtis index with various subcommunities: universal colonizers (A), ubiquitous phylotypes (B), and *Archaea* (C). The slope of each linear regression determines the spatial community shift rate (β). The linearity and the statistical significance of each model were shown using the Pearson’s correlation coefficient (ρ) and *P* value, respectively.

All three of these groups of organisms (i.e., universal colonizers, ubiquitous phylotypes, and *Archaea*) exhibited statistically significant distance decay relationships (Fig. 3). The β of the universal colonizers and of the ubiquitous phylotypes were similar to the β of the whole community. When β was calculated for each month, the β of the universal colonizers (median = 0.13) and of the ubiquitous phylotypes (median = 0.12) were not significantly different than the β of the whole community (median = 0.12) (Wilcoxon *Z *=* *0.92 and 0.32 and *P* = 0.36 and 0.75, respectively). The *Archaea* had a smaller β (0.05) compared to the whole community; the statistical significance of the distance decay relationship for the *Archaea* was weaker (*P* = 1.9 × 10^−5^) than for the whole community.

### Environmental heterogeneity and geographic distance.

Distance decay relationships can be driven by both changes in environmental conditions as well as dispersal. To better understand the relative importance of these two mechanisms, the annual average Bray-Curtis community dissimilarities between the facilities (community profiles based on genus-level phylotypes) were correlated with analogous distance matrices of facility characteristics such as geographic location, operational parameters, untreated wastewater characteristics, and treated wastewater characteristics (Table 2; raw data available in Table S1). In addition to the strong correlation between geographic location and Bray-Curtis community dissimilarities that has been previously described, the whole community dissimilarities also exhibited statistically significant correlations with total Kjeldahl nitrogen (TKN) concentrations in the treated and untreated wastewater as well as the ammonia concentrations and CBOD concentrations in the treated wastewater. The subgroups of universal colonizers and ubiquitous phylotypes generally exhibited the same correlation with the whole community, while the *Archaea* had a stronger correlation with mean cell residence time than with geographic distance.

**TABLE 2:** Spearman’s correlation coefficients (from Mantel test) between annual average environmental characteristic differences and annual average Bray-Curtis community dissimilarities or geographic distances^*a*^

Characteristic	Environmental variable^*b*^	Whole community dissimilarity	Universal colonizer dissimilarity	Ubiquitous phylotype dissimilarity	*Archaea* dissimilarity	Geographic distance
Geographic distance		0.62*	0.51*	0.61*	0.29*	

Operational parameter	Avg treatment	−0.04	0.01	−0.08	0.16	−0.11
	pH	−0.04	−0.09	−0.08	0.10	0.03
	Mean cell residence time	0.33	0.38	0.29	0.70*	0.36

Untreated wastewater characteristics	CBOD	0.18	0.19	0.19	0.10	0.15
	TSS	0.13	0.18	0.09	0.40*	0.17
	Phosphorus	0.27	0.24	0.26	0.12	0.19
	TKN	0.51*	0.47*	0.53*	0.11	0.58*

Treated wastewater characteristics	pH	0.04	−0.01	0.04	0.03	0.04
	CBOD	0.37*	0.26	0.34*	0.22	0.22
	TSS	0.19	0.24	0.21	0.17	0.16
	Phosphorus	0.24	0.22	0.31*	−0.16	0.06
	NH_3_	0.55*	0.47*	0.56*	0.13	0.43*
	NO_3_ + NO_2_	0.25	0.10	0.17	0.16	0.27
	TKN	0.64*	0.58*	0.66*	0.15	0.62*

a The arithmetic mean value was used for facilities with multiple bioreactors. The amount of data varied by facility (details in Table S1 in the supplemental material), such that *P* can vary even if the correlation coefficient is the same. An asterisk indicates statistically significant correlation (*P* < 0.001).

b CBOD, carbonaceous biochemical oxygen demand; TSS, total suspended solids; TKN, total Kjeldahl nitrogen.

Three of the environmental parameters (i.e., TKN concentrations in the treated and untreated wastewater and ammonia concentrations in the treated wastewater), which were strongly correlated with the whole community composition, also significantly correlated with geographic distance (Table 2). Partial canonical correspondence analysis (CCA) was therefore performed to separate the importance of geographic distance from facility characteristics to the whole community composition. For this analysis, only 11 of the 20 facilities participating in this study were analyzed because only these facilities provided a full set of metadata. Geographic distance explained 22.4% of the variance in microbial community composition, when environmental factors were controlled. In contrast, three environmental parameters explained 35.6% of the variance when geographic distance was controlled. Spatially structured environmental variation (i.e., the combination of geographic distance and environmental factors) explained 76.0% of the variance, which leaves 24.0% of the variance unexplained.

### Impacts of geographic scale and site selection.

Because the previous section focused on only 11 of the 20 facilities participating in this investigation (i.e., those facilities that had a full set of metadata), the effect of spatial scale on the distance decay relationships was evaluated further using community profiles based on genus-level phylotypes. That is, the partial CCA could have been biased by the specific facilities incorporated into that analysis. Specifically, seven of the facilities were located in a single metropolitan region and were of close proximity (11 km to ∼62 km; average, 30 km).

**FIG 4:**
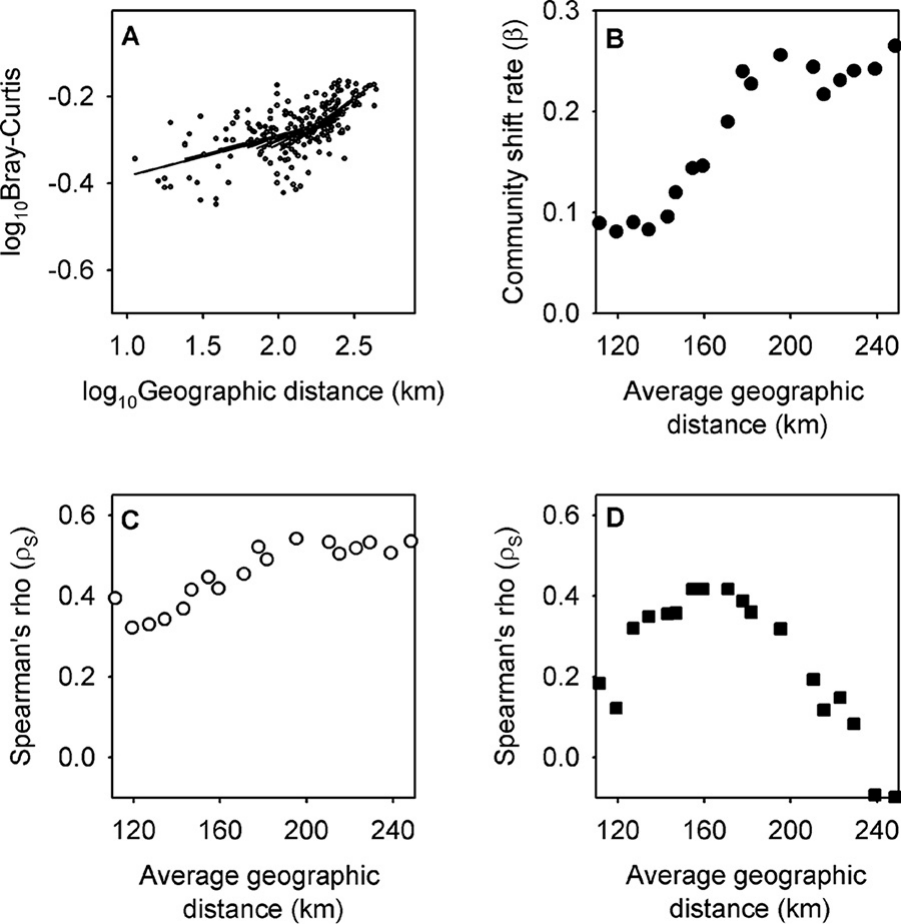
(A) Changes in spatial community shift rate (β) investigated by a sliding spatial scale (*P* < 0.001). (B) Changes in β versus average geographic distance between the facilities (i.e., the slope of the lines shown in panel A), (C) Spearman’s correlation coefficient (ρ*_s_*) between the community dissimilarity and geographic distance, and (D) ρ*_s_* between the community dissimilarity and differences in total Kjeldahl nitrogen concentrations (TKN) in the untreated wastewater. All four analyses used the geographic distance between facility pairs on a sliding scale from 10 to 210 km to 180 to 380 km, in 10-km increments, and community dissimilarity was calculated using community profiles based on genus-level phylotypes.

This study included 20 different wastewater treatment facilities, allowing 190 pairwise calculations of microbial community dissimilarities. Using a sliding window of facility pairs ranked from shortest to longest geographic distance (10-km increment each time from 10- to ∼210-km scale to 180- to ∼380-km scale), it was observed that β generally increased as the geographic scale of the study became larger (Fig. 4A and B), while all the distance decay relationships constructed within each geographic scale were statistically significant (all *P* < 0.001).

Previously, it was observed that bioreactor community dissimilarity was well correlated with both geographic distance and environmental characteristics such as TKN in the untreated wastewater. The effect of geographic-scale changes on these correlations were studied as well. The Spearman’s correlation coefficient between the bioreactor community dissimilarities and geographic distance also increased as geographic scale became bigger (Fig. 4C). In contrast, the correlation between bioreactor community dissimilarity and TKN in the untreated wastewater did not become stronger as geographic scale became bigger (Fig. 4D). This suggests that the partial CCA results reported in the previous section (i.e., bioreactor community dissimilarity was better explained by environmental heterogeneity than geographic distance) could have been biased by the specific wastewater facilities incorporated into that analysis and might not reflect the results if all 20 facilities had been analyzed.

### Seasonal changes in microbial community composition.

**FIG 5:**
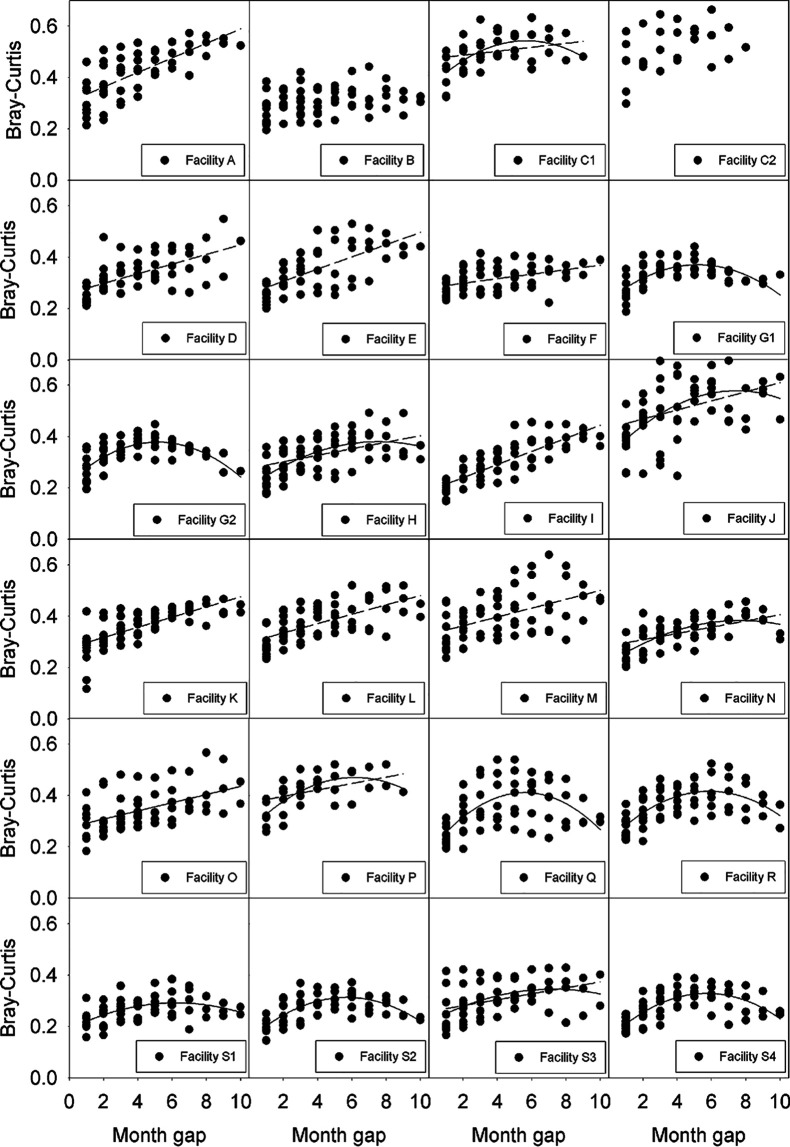
Changes in community dissimilarity as a function of the time difference between two sampling events observed in 24 activated sludge bioreactors from 19 facilities. Dashed lines indicate a statistically significant linear change in dissimilarity over time. Solid lines indicate a statistically significant fit to a quadratic regression model with an axis of symmetry between 5 and 8 months. Facility T is not shown due to a low number of samples. Community dissimilarity was calculated using community profiles using genus-level phylotypes.

We hypothesized that seasonal changes might also impact distance decay relationships. To test seasonal impacts on the microbial community composition in bioreactors (e.g., cyclic changes in beta-diversity), community composition from 24 different bioreactors (19 facilities; facility T was excluded from this analysis due to low sample numbers) was investigated as a function of the time interval between sample collection events using genus-level phylotype community profiles (Fig. 5 and Table S4). About half of the bioreactors (13 out of 24) had a statistically significant fit to a quadratic model with an axis of symmetry between 5 and 8 months, suggesting that these bioreactors had cyclic seasonal changes in microbial community composition. In contrast, about half of the bioreactors (15 out of 24) exhibited a statistically significant fit to a linear model, and two bioreactors did not exhibit a statistically significant fit to either the linear model or the quadratic model. Six of the bioreactors simultaneously had a statistically significant fit to both the quadratic and linear models.

10.1128/mSphere.00648-21.4 TABLE S4 Linear and quadratic regressions between sampling time intervals and phylotype-based community Bray-Curtis community differences. ANOVA, analysis of variance. Download Table S4, DOCX file, 0.1 MB. Copyright © 2021 Kim et al. 2021 Kim et al. https://creativecommons.org/licenses/by/4.0/ This content is distributed under the terms of the Creative Commons Attribution 4.0 International license.

Similarly, we also tested the hypothesis that distance decay relationships would be less pronounced in the winter than during the summer. When the distance decay relationships were constructed using the Bray-Curtis dissimilarities from the entire microbial community, distance decay relationships were significant (*P* < 0.001) every month (similar results were obtained for both Sørensen and weighted Unifrac indices) (Table S5), and no clear seasonal trend in β was observed (Fig. S5). Similarly, the universal colonizers and ubiquitous phylotypes also showed significant distance decay relationships for 9 and 10 months, respectively. In contrast, the *Archaea* exhibited a significant distance decay relationship during only 2 months (August 2017 and January 2018).

10.1128/mSphere.00648-21.5 TABLE S5 Statistical significance of distance decay relationships calculated each month. *P* values are from Pearson’s correlation; insignificant relationships are shown by underlined numbers. July 2017 and July 2018 are excluded since participating facilities were different. Download Table S5, DOCX file, 0.1 MB. Copyright © 2021 Kim et al. 2021 Kim et al. https://creativecommons.org/licenses/by/4.0/ This content is distributed under the terms of the Creative Commons Attribution 4.0 International license.

10.1128/mSphere.00648-21.10 FIG S5 Monthly spatial community shift rate (β) when the whole community dissimilarity was calculated with Bray-Curtis (phylotype-based) (A), Sørensen (phylotype-based), (B) and weighted Unifrac (C) indices. Monthly spatial community shift rate (β) of universal colonizers (D) and ubiquitous phylotypes (E) when calculated with Bray-Curtis dissimilarity indices. Distance decay relationships that were not statistically significant were excluded (Pearson’s correlation *P* ≥ 0.001; *P* values are tabulated in Table S5). Download FIG S5, DOCX file, 0.1 MB. Copyright © 2021 Kim et al. 2021 Kim et al. https://creativecommons.org/licenses/by/4.0/ This content is distributed under the terms of the Creative Commons Attribution 4.0 International license.

## DISCUSSION

Understanding the spatiotemporal patterns of biodiversity is a central goal of ecology. The distance decay of community similarity is one of the spatial scaling patterns observed in many forms of life, including plants (2, 4), animals (1, 4), and microbial communities (4, 9–11). However, relatively few studies have explored distance decay relationships in municipal wastewater treatment bioreactors (17, 18). Municipal wastewater treatment is critically important for protecting surface water quality; it relies on microorganisms to reduce the release of excessive nutrients (biodegradable organic carbon, nitrogen, and phosphorus) as well as other priority pollutants to the environment. Within the studied geographic scale (<442 km), a significant distance decay pattern was observed among microbial communities in wastewater treatment bioreactors. These distance decay relationships were also robust, exhibiting statistical significance regardless of the sequence clustering method or the community dissimilarity metric. Our results strongly suggest that microbial communities in wastewater treatment bioreactors are not randomly assembled but rather exhibit a spatial pattern. This novel understanding of the mechanisms of microbial community assembly in wastewater treatment bioreactors builds on prior work that demonstrated synchrony in wastewater treatment bioreactor communities in close proximity to each other (19) and weak distance decay relationships over much larger spatial scales (∼3,000 km) (17).

The distance decay of community composition can be explained by two different factors: gradients in environmental conditions and dispersal limitation. Our research makes a novel contribution by demonstrating that environmental heterogeneity is a more important driver of the observed distance decay relationships than dispersal limitation in wastewater treatment bioreactors. Distinguishing these two mechanisms is challenging because differences in environmental conditions are generally confounded by geographic distance (21). Universal colonizers (detected in all bioreactors during all sampling times) and ubiquitous phylotypes (detected in all facilities on at least one occasion) were studied in more detail because we hypothesized that these organisms were unaffected by dispersal limitation. Both subgroups exhibited strong distance decay relationships when calculated using Bray-Curtis dissimilarity, demonstrating that environmental gradients were sufficient to produce a statistically significant distance decay relationship. Furthermore, the β values for both subgroups were approximately the same as the β value for the entire microbial community, suggesting that environmental gradients were the dominant factor for the observed distance decay relationship of microbial communities in wastewater treatment bioreactors. Previous researchers concluded that environmental filtering is a more important driver of biogeographic patterns at smaller spatial scales and dispersal limitation is important at large-scale spatial patterns (11, 22); it is possible, therefore, that the distances studied herein (<442 km) are insufficient to observe a stronger dispersal limitation. That is, at least for this distance scale, “everything is more or less everywhere, but the environment selects” (23–25).

We hypothesized that the distance decay relationship of the archaeal community would largely be driven by dispersal rather than an active response to environmental conditions. Previous research has demonstrated that only a small amount of methanogenic activity occurs in full-scale activated sludge bioreactors, resulting in only a minor fraction of the overall carbon cycling (26). The majority of the *Archaea* detected in our study were methanogens that are well-known to be obligate anaerobes; ammonia-oxidizing archaea were not detected. Although the archaeal community exhibited a statistically significant distance decay relationship, the β for the archaeal community was much smaller than the β for universal colonizers and ubiquitous phylotypes. This suggests that the effect of the dispersal (β = 0.05) is substantially smaller than environmental heterogeneity (β = 0.12 to 0.13) in the distance decay relationships at this scale.

Partial CCA also suggested that environmental heterogeneity was a more important factor in the observed distance decay relationships than dispersal limitation. This is consistent with previous researchers who concluded that bacterial community assembly in wastewater treatment bioreactors is controlled by deterministic (i.e., niche-based community assembly) rather than stochastic mechanisms (19). However, quantitative correlations can change based on geographic scale and/or site selection. In this study, bioreactor community dissimilarity was more strongly correlated with geographic distance as geographic distance increased. In contrast, the correlation between community dissimilarity and the TKN in the untreated wastewater exhibited a hump-shaped profile as geographic distance increased. That is, there is an incongruity between the mathematical importance of TKN in the untreated wastewater, which had a strong Spearman’s correlation with community dissimilarity, and bioreactor community dissimilarity as distance increases. Admittedly, our analysis is implicitly limited by the number of environmental parameters considered; numerous other deterministic factors (e.g., concentrations of specific chemicals) were not considered in our CCA.

Our experimental design (20 facilities; 12 months) afforded us the opportunity to observe temporal changes in community richness and temporal changes in distance decay relationships. Previous studies have found that bioreactors showed seasonal cyclic patterns in alpha-diversity, with higher diversity (or richness) during warmer seasons and lower diversity during colder seasons (19, 27). However, cyclic fluctuations in community alpha-diversity driven by seasonal changes were not observed and alpha-diversity did not correlate with the monthly average temperature in our study. Similarly, the distance decay relationships for the entire microbial community, the universal colonizers, and the ubiquitous phylotypes were statistically significant as a function of time. This suggests that the distance decay relationships observed herein are robust and consistent with deterministic mechanisms of community assembly.

Our experimental design also afforded us the opportunity to observe cyclical patterns in microbial community composition at numerous wastewater treatment facilities over the same time period. Previous studies have shown cyclic seasonal patterns in beta-diversity (19, 28, 29); that is, sample pairs collected from the same bioreactor showed increasing community dissimilarity with increasing time gaps for ∼7 months but then subsequently showed decreasing dissimilarity (19, 28). However, these claims may have been spurious because of the relatively small number of facilities (<5) and the short durations (<14 months) of these studies. In our study, seasonal variations in community composition (i.e., a cyclic pattern in beta-diversity) was observed in about half of the bioreactors, similar to the number of bioreactors that exhibited a linear (i.e., nonreturning) pattern in community dissimilarity. This suggests that the cyclic community changes may be a site-specific or spurious observation. Additional research is needed for longer time periods to properly address this question.

A statistically significant taxon-volume relationship was found between the number of observed OTUs and the relative size of the treatment facilities based on annual average flow rate. This suggests that island biogeography theory, which predicts more species in larger ecosystems (30), may also apply to municipal wastewater treatment bioreactors. A previous study made a similar observation during the investigation of membrane bioreactors used for municipal wastewater (31). Both taxon-volume relationships and distance decay relationships are pertinent ecological theories that explain spatial biodiversity patterns; several studies have predicted that the taxon-area relationship exponent *z* would be equal to one-half the spatial community shift rate β, as calculated via the Sørensen index (6, 15, 17, 32). In our study, however, *z* and β did not correlate as predicted. Furthermore, genus-level phylotype-based community profiles did not exhibit a statistically significant taxon-volume relationship, whereas a strong distance decay relationship was observed. Further research is needed to reconcile the relationships between island biogeography and distance decay in controlling microbial community composition in municipal wastewater treatment bioreactors.

Although we report values for β, we caution against comparing these values with those from past and future studies. Specifically, biodiversity patterns vary systematically as a function of the taxonomic resolution used to describe microbial community composition (33). Even this conclusion is controversial because a previous study concluded that the OTU similarity cutoff did not impact β in soil samples (11), whereas another study found that narrowing the similarity cutoff increased *z* (estimated from β) in salt marsh sediments (15). In our study, narrowing the similarity cutoff (97% sequences base similarity [OTU] versus genus-level phylotypes) effectively reduced β in wastewater treatment bioreactors. In addition, removing rare sequences (or low-abundance OTUs) affected β (34) as did changing sequencing depth (11). Previous studies suggested that different spatial scales also can affect β (1, 18, 35); our results support these conclusions because β increased as the spatial scale became larger in the range of 10 to 380 km.

In conclusion, this study showed that microbial community composition exhibited a strong distance decay relationship in community similarity in municipal wastewater treatment facilities within 442 km of each other. Our results suggest that environmental heterogeneity is a more important factor than dispersal limitation, which strongly implies that deterministic community assembly mechanisms dominate municipal wastewater treatment bioreactors. Our results demonstrate a significant effect of geographic scale on distance decay relationships, suggesting that caution should be used before generalizing our quantitative results. We observed that the ecological theory that had been developed for plants and animals could be applied to microbial communities in engineered systems. Additional research is needed to identify and understand how specific factors affect specific groups of microorganisms during municipal wastewater treatment.

## MATERIALS AND METHODS

### Wastewater treatment facility description.

Twenty wastewater treatment facilities (facility A to facility T) in the state of Minnesota participated in this study. All the facilities utilize continuous aeration and recirculation of settled/concentrated biomass with some variation in process design and operation (see Table S2 in the supplemental material). Facilities with unique system designs (e.g., adsorption/bio-oxidation systems, membrane bioreactors, and sequencing batch reactors) were purposely excluded from this study because such system designs could confound the interpretation of spatial biogeographic patterns. Facilities C and G were equipped with two independent parallel bioreactors (C1 and C2 and G1 and G2), and facility S had four parallel bioreactors (S1 to S4). Untreated and treated wastewater characteristics were measured at each facility (Table S2). Each of the laboratories at the wastewater treatment facilities are certified by the U.S. EPA, and all of their methods are described in *Standard Methods for the Examination of Water and Wastewater* (36).

### Sample collection and DNA extraction.

Grab samples (volume, 10 to 15 ml) were collected by facility operators on the same week and kept frozen until processed. Samples were collected 12 times (approximately monthly) from July 2017 until July 2018. Aliquots of bioreactor samples (0.1 ml) were mixed with 0.9 ml of lysis buffer (5% sodium dodecyl sulfate, 120 mM sodium phosphate buffer [pH 8]). Mixed samples underwent three freeze-thaw cycles followed by 90 min of incubation at 70°C. DNA was then purified using the FastDNA kit (MP Biomedicals, Santa Ana, CA, USA) per the manufacturer’s instructions.

### Amplicon sequencing.

From each sample, amplicons of the V4 region of the 16S rRNA gene were generated using primer set Meta_V4_515F and Meta_V4_806R and subsequently sequenced using Illumina MiSeq at the University of Minnesota Genomics Center as described previously (37). Each MiSeq run was loaded with a pooled library of amplicons (8 pM) with 15% PhiX. Cluster densities ranged from 824 to 1,104 K mm^−2^; 83.6% ± 5.2% of the bases had quality scores higher than 30.

### DNA sequence analysis.

For sequence analysis, mothur (version 1.40.0) was used (38), with a protocol similar to the MiSeq standard operating procedure (SOP) (39). Paired-end reads were merged, and sequences with one or more ambiguous bases or with a homopolymer longer than 8 nucleotides were removed. Sequences were aligned to SILVA v.128 reference file. Aligned sequences were filtered to remove columns that contained gaps and sequences with more than 300 bases or fewer than 280 bases were discarded. Chimeric sequences were identified and discarded with Vsearch (40). Sequences were classified with the Bayesian classifier using RDP v.16 reference files (41); sequences that did not classify as *Bacteria* or *Archaea* were discarded (i.e., unknown, chloroplast, mitochondria, or Eukaryota). OTU-based analysis was done using 4,336 sequences per sample to simultaneously maximize the number of microbiome profiles analyzed and the depth of sequences per microbiome profile. For OTU-based analysis, the sequences were clustered into OTUs at 97% similarity using the OptiClust method (42). For phylotype-based analysis, sequences were binned into phylotypes according to their genus-level taxonomic classification. Genus-level phylotypes show communities at a lower resolution (i.e., less richness and diversity) than OTUs; therefore, more sequences per sample were used. Phylotype-based analysis was performed using 7,805 sequences per sample in an attempt to optimize the number of qualifying microbiome profiles and sequencing depth. A phylogenetic tree was constructed with clear-cut using 1,000 random sequences per sample (43). AMOVA was performed to determine whether there was a significant difference in the microbial community composition in different bioreactors (44–46).

### Data analysis.

The taxon-volume relationship exponent *z* was calculated using the following equation (47):

$${\text{log}}_{10}\left(R\right)=\;z{\text{ log}}_{10}\left(V\right)\;+\;c$$

where *R* is community richness (e.g., the number of observed OTUs) in each bioreactor, *V* is the flow rate of the bioreactor, and *c* is an empirically derived constant. The spatial community shift rate (β) was calculated using the following equation (2):

$${\text{log}}_{10}\left(\mathrm{DS}\right)={\text{ β log}}_{10}\left(D\right)\;+\;c$$

where *DS* is the community dissimilarity between two bioreactors, *D* is the geographic distance between two bioreactors, and *c* is an empirically derived constant. Linear least-squares regressions were performed to estimate *z* and β. The arithmetic means from all the possible bioreactor pairs were used when dissimilarity calculation includes facilities with multiple bioreactors (e.g., dissimilarity between facilities B and C was calculated as the arithmetic mean of B-C1 and B-C2 dissimilarities). The linearity of both models was tested using Pearson’s linear correlation.

Spearman’s rank correlation was used to determine whether two parameters had a monotonic relationship. Two-tailed Wilcoxon rank sum tests were used to determine whether a metric for one group was significantly different than another group. Linear and quadratic regressions were performed using the time interval between two sampling events versus community dissimilarity (phylotype-based); analysis of variance (ANOVA) and axis of symmetry were then tested to determine which bioreactors exhibited linear and/or cyclic changes in community composition. Community dissimilarities were correlated with geographic distance and environmental differences (operational parameters and wastewater characteristics) using Mantel tests with Spearman’s rank correlation. Using variables identified as having statistically significant correlations with community dissimilarities, CCA was performed to estimate the fraction that each environmental variable explained community dissimilarities. Mantel tests and CCA were performed using the package “vegan” in R (48); the significance of the CCA model was determined by analysis of variance (ANOVA) with 999 permutations. All the other statistical analyses were performed using MATLAB R2019a (MathWorks, Natick, MA, USA).

A significance level of α = 0.001 was assumed throughout this study. This value was selected because the relatively high number of microbiome profiles (*n *=* *190) could lead to “statistically significant” results even when the correlations were not strong; in addition, computed correlation coefficients and *P* values were reported throughout.

### Accession number(s).

The sequences of this study have been deposited in NCBI Sequence Read Archive under accession number PRJNA560576.
