# Supplementary material for: Microbial Community Composition in Municipal Wastewater Treatment Bioreactors Follows a Distance Decay Pattern Primarily Controlled by Environmental Heterogeneity
Source: mSphere. 2021 Oct 20;6(5):e00648-21. doi: 10.1128/mSphere.00648-21 (PMC8527990; doi:10.1128/mSphere.00648-21)
Supplement: TABLE S1 [file msphere.00648-21-st001.docx]

**Table S1.** Annual average operational parameters (OTU: operational taxonomic unit, CBOD: carbonaceous biochemical oxygen demand, TSS: total suspended solids, TKN: total Kjeldahl nitrogen; mg/L for unmarked units)

|  |  | A | B | C | D | E | F | G | H | I | J | K | L | M | N | O | P | Q | R | S | T |
| --- | --- | --- | --- | --- | --- | --- | --- | --- | --- | --- | --- | --- | --- | --- | --- | --- | --- | --- | --- | --- | --- |
| Community richness | #observed phylotypes | 269.7 | 278 | 226.2 | 285 | 261.5 | 238.6 | 275.1 | 267.7 | 248.6 | 239.8 | 279.5 | 267.6 | 239.7 | 276.8 | 255.6 | 261.3 | 269.8 | 275 | 265.4 | 260.2 |
|  | #observed OTUs | 2082 | 2173 | 1375 | 1911 | 2166 | 1392 | 2052 | 2196 | 2012 | 1975 | 1968 | 2072 | 1986 | 2196 | 2172 | 1921 | 2239 | 2204 | 2230 | 2068 |
| Operational parameter | Flow-rate (megaliter/day) | 17 | 0.9 | 4.5 | 3.8 | 16.7 | 45.4 | 84.8 | 37.9 | 9.1 | 5.7 | 3.8 | 34.1 | 12.1 | 1.1 | 53 | 3.4 | 110.2 | 26.5 | 660.2 | 0.8 |
|  | pH | 7.6 | 7.7 | 7.6 | 8.1 | 7.4 | 7.1 | 7.4 | 7.7 | 7.7 | 7.5 | 7.4 | 7.3 | 7.6 |  |  | 7.7 | 7.1 | 7 |  |  |
|  | Mean cell residence time (days) |  |  | 8.1 |  | 8.9 | 0.9 | 11 | 7.9 | 80 | 9.9 | 11.7 |  |  |  |  | 20 |  |  | 11.9 |  |
| Untreated wastewater | CBOD | 278.2 | 1230 | 411.4 | 153.7 | 167.5 | 185.2 | 178.3 |  | 264.6 | 182.5 | 144.9 | 168.3 | 60.7 |  |  | 163.1 | 190.1 | 205.2 | 143.8 |  |
|  | TSS | 1918 | 5220 | 2176 | 432 | 2570 | 2792 | 2455 | 2549 | 8114 | 2347 | 141 | 235 | 1933 |  |  | 195 | 2941 | 235 | 2344 |  |
|  | Phosphorus |  | 15.4 | 10.3 | 3.6 | 6 | 2.5 | 8.1 | 6.6 | 4.8 | 5.9 | 3.3 | 5.7 | 2 |  |  | 7.8 | 7.5 | 5.2 | 5.3 |  |
|  | TKN |  |  | 72.9 |  | 48.3 | 20.4 | 39.8 | 43.8 |  | 43.3 | 22.3 |  | 19.6 |  |  |  | 48.4 | 41.5 | 38.3 |  |
| Treated wastewater | pH |  |  | 7 |  | 7.1 | 6.9 | 7.3 | 7.2 | 7.4 | 6.9 | 5.6 | 7 | 6.8 |  |  | 7.1 | 7 | 6.7 | 7.1 |  |
|  | CBOD |  | 1.1 | 3.2 | 0.9 | 3.7 | 6.3 | 3.5 | 2.8 | 1.8 | 6.4 | 4.2 | 1.7 | 3.3 |  |  | 2.1 | 2.4 | 3.4 | 3.9 |  |
|  | TSS |  | 5 | 2.8 | 8 | 3.6 | 4.4 | 5.6 | 3.5 | 9.7 | 7.9 |  | 5.2 | 3.7 |  |  | 10.6 | 3.3 | 3.2 | 6 |  |
|  | Phosphorus |  | 0.4 | 0.1 | 0.8 | 0.3 | 0.4 | 0.5 | 0.8 | 0.6 | 3.7 | 1.8 | 0.3 | 0.4 |  |  | 0.7 | 0.4 | 0.2 | 0.4 |  |
|  | NH_3_ |  |  | 16.1 | 0.2 | 0.1 | 8.2 | 0.7 | 0.5 | 0.1 | 2.9 | 7.9 | 0.2 | 0 |  |  | 0.1 | 1.9 | 0.3 | 5.2 |  |
|  | NO_3_ + NO_2_ |  |  | 19.3 |  | 25.2 | 0.2 | 18.4 | 26.7 | 18.3 | 24.3 | 6.7 |  | 18.1 |  |  |  | 22.3 | 9.5 | 15.8 |  |
|  | TKN |  |  | 21.3 |  | 1.9 | 10.8 | 2.8 | 2.2 | 1.9 | 5.9 | 9.7 |  | 1.5 |  |  |  | 3.6 | 2.1 | 6.7 |  |
