# Supplementary material for: Microbial Community Composition in Municipal Wastewater Treatment Bioreactors Follows a Distance Decay Pattern Primarily Controlled by Environmental Heterogeneity
Source: mSphere. 2021 Oct 20;6(5):e00648-21. doi: 10.1128/mSphere.00648-21 (PMC8527990; doi:10.1128/mSphere.00648-21)
Supplement: TABLE S2 [file msphere.00648-21-st002.docx]

**Table S2.** Facility characteristics and design components

|  | Component |
| --- | --- |
| A | Grit Removal, Preaeration - less than two hours detention time, Primary Clarifier, Activated Sludge (contact stabilization, conventional, step feed), Secondary Clarifier, Tertiary Clarifier, Filter -sand with backwash, Ultraviolet Light, Anaerobic Digester - complete mixed, heated - mesophilic, Dissolved Air Flotation, Rotary Drum Thickening, Storage Tank |
| B | Collection with gravity and/or Pressure Sewer, Influent Flow Meter, Fine Screen, Bar Screen - manual, Grit Removal, Phosphorus Removal - biological, Activated Sludge - extended aeration, Phosphorus Removal - chemical, Secondary Clarifier, Chlorination, Dechlorination, Postaeration - less than two hours, detention time, Storage Tank, Gravity Thickening, Storage Tank, Land Application |
| C | Bar Screen - mechanical, Grit Removal, Primary Clarifier, Activated Sludge - contact stabilization, conventional, step feed, Phosphorus Removal - chemical, Secondary Clarifier, Filter - sand with backwash, Ultraviolet Light, Anaerobic Digester – complete mixed heated - mesophilic, Storage Tank |
| D | Bar Screen - mechanical, Grit Removal, Fine Screen, Flow Equalization, Primary Clarifier, Trickling Filter, Trickling Filter - solids contact, Phosphorus Removal - chemical, Secondary Clarifier, Effluent Polishing Filter with backwash, Anaerobic Digester - complete mixed, heated - mesophilic, Storage Tank, Chlorination, Dechlorination, Activated Sludge (contact stabilization, conventional, step feed) |
| E | Activated Sludge (contact stabilization, conventional, step feed), Anaerobic Digester (complete mixed, heated – mesophilic), Bar Screen - mechanical, Chlorination, Dechlorination, Gravity Thickening, Secondary Clarifier |
| F | Collection with gravity and/or Pressure Sewer, Bar Screen - mechanical, Grit Removal, Activated Sludge - pure oxygen, Secondary Clarifier, Flocculation, Filter - sand with backwash, Chlorination, Dechlorination, Dissolved Air Flotation, Anaerobic Digester - complete mixed, heated - thermophilic, Anaerobic Digester (complete mixed, heated – mesophilic), Storage Tank, Centrifuge, Storage Tank, Land Application |
| G | Collection with gravity and/or Pressure, Sewer, Bar Screen - mechanical, Grit Removal, Primary Clarifier, Activated Sludge (contact stabilization, conventional, step feed), Secondary Clarifier, Phosphorus Removal - biological, Phosphorus Removal - chemical, Chlorination, Dechlorination, Postaeration (less than two hours), detention time, Storage Tank, Dissolved Air Flotation, Centrifuge, Centrifuge, Incineration |
| H | Collection with gravity and/or Pressure, Sewer, Flow Equalization, Fine Screen, Grit Removal, Primary Clarifier, Anaerobic Contactor, Phosphorus Removal - biological, Activated Sludge (contact stabilization, conventional, step feed), Phosphorus Removal - chemical, Secondary Clarifier, Ultraviolet Light, Effluent Pumping, Gravity Thickening, Gravity Thickening, Anaerobic Digester (complete mixed, heated, mesophilic), Belt Filter Press, Storage Tank |
| I | Grit Removal, Activated Sludge (contact stabilization, conventional, step feed), Influent Flow Meter, Bar Screen - mechanical, Anaerobic Digester (complete mixed, heated – mesophilic), Heat Drying - indirect or direct heat, Belt Filter Press, Gravity Belt, Secondary Clarifier, Ultraviolet Light, Primary Clarifier, Land Application, Phosphorus Removal - chemical, Dewatered Sludge Storage Pad/Shed |
| J | Collection with gravity and/or Pressure, Sewer, Bar Screen - mechanical, Grit Removal, Primary Clarifier, Activated Sludge (contact stabilization, conventional, step feed), Secondary Clarifier, Chlorination, Dechlorination, Gravity Thickening, Anaerobic Digester (complete mixed, heated, mesophilic) |
| K | Primary Clarifier, Activated Sludge (contact stabilization, conventional, step feed), Flow Equalization, Bar Screen - mechanical, Centrifuge, Dewatered Sludge Storage Pad/Shed, Aerobic Storage Tank, RDP - Lime plus Pasteurization, Grit Removal, Chlorination, Secondary Clarifier |
| L | Activated Sludge (contact stabilization, conventional, step feed), Gravity Thickening, Dissolved Air Flotation, Anaerobic Digester (complete mixed, heated – mesophilic), Grit Removal, Flow Equalization, Chlorination, Dechlorination, Secondary Clarifier, Primary Clarifier |
| M | Collection with gravity and/or Pressure, Sewer, Bar Screen - mechanical, Grit Removal, Primary Clarifier, Activated Sludge (contact stabilization, conventional, step feed), Secondary Clarifier, Phosphorus Removal - chemical, Ultraviolet Light, Gravity Thickening, Storage Tank |
| N | Bar Screen - mechanical, Influent Flow Meter, Pumping (Lift) Station, Grit Removal, Primary Clarifier, Activated Sludge (contact stabilization, conventional, step feed), Phosphorus Removal - chemical, Secondary Clarifier, Effluent Flow Meter, Chlorination, Dechlorination, Storage Tank, Anaerobic Digester (complete mixed, heated – mesophilic), Storage Tank, Land Application |
| O | Activated Sludge - contact stabilization, conventional, step feed, Anaerobic Digester (complete mixed, heated – mesophilic), Bar Screen - mechanical, Centrifuge, Chlorination, Dechlorination, Flow Equalization, Grit Removal, Phosphorus Removal - chemical, Preaeration - less than two hours, detention time, Secondary Clarifier |
| P | Fine Screen, Grit Removal, Activated Sludge - extended aeration, Secondary Clarifier, Ultraviolet Light, Phosphorus Removal - chemical, Postaeration - less than two hours, detention time, Dissolved Air Flotation, Storage Tank, Lime Treatment (Class B), Land Application |
| Q | Bar Screen - mechanical, Grit Removal, Primary Clarifier, Phosphorus Removal - biological, Activated Sludge (contact stabilization, conventional, step feed), Secondary Clarifier, Aerated Polishing Ponds - less than 180, days detention time, Chlorination, Dechlorination, Postaeration - less than two hours, detention time, Postaeration - less than two hours, detention time, Gravity Thickening, Belt Filter Press, Gravity Belt, Centrifuge, Heat Drying - indirect or direct heat, Pelletization |
| R | Collection with gravity and/or Pressure, Sewer, Bar Screen - mechanical, Influent Flow Meter, Bar Screen - manual, Bar Screen - mechanical, Grit Removal, Primary Clarifier, Activated Sludge (contact stabilization, conventional, step feed), Secondary Clarifier, Phosphorus Removal - biological, Phosphorus Removal - chemical, Ultraviolet Light, Gravity Thickening, Anaerobic Digester (complete mixed, heated – mesophilic), Storage Tank, Storage Tank, Land Application, Effluent Pumping, Effluent Screen, Chlorination |
| S | Activated Sludge (contact stabilization, conventional, step feed), Belt Filter Press, Gravity Belt, Chlorination, Dechlorination, Dissolved Air Flotation, Gravity Thickening, Grit Removal, Incineration, Phosphorus Removal – biological, Rotating Biological Surfaces, Secondary Clarifier |
| T | Pumping (Lift) Station, Fine Screen, Grit Removal, Activated Sludge - contact stabilization, conventional, step feed, Secondary Clarifier, Ultraviolet Light, Storage Tank |
