# Supplementary material for: Microbial Community Composition in Municipal Wastewater Treatment Bioreactors Follows a Distance Decay Pattern Primarily Controlled by Environmental Heterogeneity
Source: mSphere. 2021 Oct 20;6(5):e00648-21. doi: 10.1128/mSphere.00648-21 (PMC8527990; doi:10.1128/mSphere.00648-21)
Supplement: FIG S1 [file msphere.00648-21-sf001.docx]

**FIG S1.** Approximate relative geographic distances between 20 facilities, plotted using longitude and latitude. Distances between the facilities ranged from 11-km to 442-km.
