# Supplementary material for: Microbial Community Composition in Municipal Wastewater Treatment Bioreactors Follows a Distance Decay Pattern Primarily Controlled by Environmental Heterogeneity
Source: mSphere. 2021 Oct 20;6(5):e00648-21. doi: 10.1128/mSphere.00648-21 (PMC8527990; doi:10.1128/mSphere.00648-21)
Supplement: FIG S2 [file msphere.00648-21-sf002.docx]

**FIG S2.** Average flow-rate and (A) the number of observed OTUs (operational taxonomic units) and (B) the number of observed phylotypes in log-scale. The taxa-volume relationship exponents (z; the slope from linear regression), the correlation coefficients (Pearson’s ρ), and the *P*-values are shown in each figure.
