# Supplementary material for: Microbial Community Composition in Municipal Wastewater Treatment Bioreactors Follows a Distance Decay Pattern Primarily Controlled by Environmental Heterogeneity
Source: mSphere. 2021 Oct 20;6(5):e00648-21. doi: 10.1128/mSphere.00648-21 (PMC8527990; doi:10.1128/mSphere.00648-21)
Supplement: TABLE S3 [file msphere.00648-21-st003.docx]

**Table S3**. Spearman’s partial correlation coefficients and *P*-values between the number of observed OTUs and three environmental variables. * indicates statistically significant correlation (*P* < 0.001). (CBOD: carbonaceous biochemical oxygen demand)

| Spearman’s ρ | Average flow-rate | Mean cell residence time | CBOD |
| --- | --- | --- | --- |
| Without control | 0.39* | 0.37* | -0.32* |
| Control average flow- rate | - | 0.23 | -0.21 |
| Control mean cell residence time | 0.51* | - | -0.48* |
| Control CBOD | 0.52* | 0.30* | - |
|  |  |  |  |
| *P* | Average flow-rate | Mean cell residence time | CBOD |
| Without control | 1.2× 10^-11^ | 4.0 ×10^-6^ | 8.7 × 10^-7^ |
| Control average flow- rate | - | 0.004 | 0.002 |
| Control mean cell residence time | 3.4 × 10^-11^ | - | 3.1 × 10^-9^ |
| Control CBOD | 9.8 × 10^-17^ | 3.8 × 10^-4^ | - |
