# Supplementary material for: Microbial Community Composition in Municipal Wastewater Treatment Bioreactors Follows a Distance Decay Pattern Primarily Controlled by Environmental Heterogeneity
Source: mSphere. 2021 Oct 20;6(5):e00648-21. doi: 10.1128/mSphere.00648-21 (PMC8527990; doi:10.1128/mSphere.00648-21)
Supplement: FIG S3 [file msphere.00648-21-sf003.docx]

**FIG S3.** Bray-Curtis dissimilarities between all the samples (phylotype-based community profile) visualized with Principal Coordinate Analysis (PCoA).
