# Supplementary material for: Microbial Community Composition in Municipal Wastewater Treatment Bioreactors Follows a Distance Decay Pattern Primarily Controlled by Environmental Heterogeneity
Source: mSphere. 2021 Oct 20;6(5):e00648-21. doi: 10.1128/mSphere.00648-21 (PMC8527990; doi:10.1128/mSphere.00648-21)
Supplement: FIG S4 [file msphere.00648-21-sf004.docx]

**FIG S4.** (A) Number of bioreactors that were colonized by each phylotype (sorted by abundance rank) and (B) number of bioreactors that each phylotypes were found. The correlation coefficients between phylotype abundance rank and number of bioreactors (Spearman’s ρ_s_) and the *P*-values are shown in each figure. Y=25 at panel A were categorized as universal colonizer, and Y=25 at panel B were categorized ubiquitous phylotypes.
