# Supplementary material for: Microbial Community Composition in Municipal Wastewater Treatment Bioreactors Follows a Distance Decay Pattern Primarily Controlled by Environmental Heterogeneity
Source: mSphere. 2021 Oct 20;6(5):e00648-21. doi: 10.1128/mSphere.00648-21 (PMC8527990; doi:10.1128/mSphere.00648-21)
Supplement: TABLE S4 [file msphere.00648-21-st004.docx]

**Table S4.** Linear and quadratic regressions between sampling time intervals and phylotype-based community Bray-Curtis community differences (ANOVA: analysis of variance)

| Facility | Linear regression ANOVA *P* | Linear regression Chi-square *P* | Quadratic regression  ANOVA *P* | Quadratic regression  Chi-square *P* | Quadratic regression axis of symmetry |
| --- | --- | --- | --- | --- | --- |
| A | <0.0001 | 0.62 | <0.0001 | 0.88 | 10.5 |
| B | 0.0427 | 0.77 | 0.0448 | 0.14 | 6.7 |
| C1 | 0.0009 | 0.22 | <0.0001 | 0.98 | 5.7 |
| C2 | 0.0419 | 0.85 | 0.0342 | 1.00 | 5.3 |
| D | <0.0001 | 0.56 | <0.0001 | 0.60 | 10.6 |
| E | <0.0001 | 0.64 | <0.0001 | 0.99 | 8.2 |
| F | 0.0009 | 0.92 | 0.0034 | 0.90 | 11.4 |
| G1 | 0.0785 | 0.08 | <0.0001 | 0.55 | 5.2 |
| G2 | 0.0719 | 0.07 | <0.0001 | 0.93 | 5.2 |
| H | <0.0001 | 0.39 | <0.0001 | 0.98 | 7.6 |
| I | <0.0001 | 0.45 | <0.0001 | 1.00 | 10.5 |
| J | <0.0001 | 0.52 | <0.0001 | 0.96 | 7.4 |
| K | <0.0001 | 0.34 | <0.0001 | 0.98 | 8.8 |
| L | <0.0001 | 0.53 | <0.0001 | 0.92 | 8.9 |
| M | <0.0001 | 0.97 | <0.0001 | 1.00 | 8.9 |
| N | <0.0001 | 0.01 | <0.0001 | 0.11 | 7.7 |
| O | <0.0001 | 1.00 | <0.0001 | 1.00 | 44.5 |
| P | 0.0001 | 0.18 | <0.0001 | 0.92 | 6.2 |
| Q | 0.0229 | 0.01 | <0.0001 | 0.94 | 5.6 |
| R | 0.0013 | 0.01 | <0.0001 | 1.00 | 5.9 |
| S1 | 0.002 | 0.23 | <0.0001 | 0.99 | 6.2 |
| S2 | 0.0017 | 0.00 | <0.0001 | 0.95 | 5.7 |
| S3 | 0.0005 | 0.83 | 0.0003 | 1.00 | 7.3 |
| S4 | 0.0013 | 0.00 | <0.0001 | 0.88 | 5.7 |
