# Supplementary material for: Microbial Community Composition in Municipal Wastewater Treatment Bioreactors Follows a Distance Decay Pattern Primarily Controlled by Environmental Heterogeneity
Source: mSphere. 2021 Oct 20;6(5):e00648-21. doi: 10.1128/mSphere.00648-21 (PMC8527990; doi:10.1128/mSphere.00648-21)
Supplement: TABLE S5 [file msphere.00648-21-st005.docx]

**Table S5.** Statistical significance of distance decay relationships calculated each month (*P*-values from Pearson’s correlation; insignificant relationships are shown with underlined number; July 2017 and July 2018 are excluded since participating facilities were different)

|  | Whole community  (Bray-Curtis) | Whole community (Sørensen) | Whole community (Weighted Unifrac) | Universal colonizers | Ubiquitous phylotypes | *Archaea* |
| --- | --- | --- | --- | --- | --- | --- |
| Aug-17 | 3.3 × 10^-4^ | 6.8 × 10^-14^ | 2.5 × 10^-4^ | 0.004 | 0.013 | 2.9 × 10^-4^ |
| Sep-17 | 3.1 × 10^-10^ | 5.3 × 10^-21^ | 7.2 × 10^-11^ | 4.9 × 10^-4^ | 1.1 × 10^-6^ | 0.011 |
| Oct-17 | 2.8 × 10^-16^ | 1.3 × 10^-23^ | 2.2 × 10^-16^ | 1.0 × 10^-6^ | 2.0 × 10^-15^ | 0.003 |
| Nov-17 | 7.2 × 10^-15^ | 9.3 × 10^-15^ | 2.1 × 10^-15^ | 8.8 × 10^-9^ | 2.2 × 10^-13^ | 0.323 |
| Dec-17 | 1.8 × 10^-11^ | 4.3 × 10^-19^ | 5.6 × 10^-14^ | 1.3 × 10^-6^ | 4.0 × 10^-9^ | 0.029 |
| Jan-18 | 4.1 × 10^-19^ | 3.9 × 10^-30^ | 1.9 × 10^-17^ | 2.6 × 10^-8^ | 4.3 × 10^-14^ | 2.7 × 10^-4^ |
| Feb-18 | 3.3 × 10^-16^ | 1.7 × 10^-16^ | 4.6 × 10^-11^ | 1.2 × 10^-7^ | 9.5 × 10^-15^ | 0.005 |
| Mar-18 | 1.8 × 10^-12^ | 3.5 × 10^-21^ | 2.7 × 10^-10^ | 1.3 × 10^-6^ | 1.0 × 10^-10^ | 0.057 |
| Apr-18 | 1.5 × 10^-11^ | 1.5 × 10^-23^ | 1.3 × 10^-7^ | 0.029 | 6.4 × 10^-9^ | 0.023 |
| May-18 | 1.2 × 10^-18^ | 1.3 × 10^-20^ | 3.5 × 10^-18^ | 1.9 × 10^-10^ | 9.8 × 10^-18^ | 0.019 |
| Jun-18 | 1.1 × 10^-20^ | 5.6 × 10^-15^ | 1.5 × 10^-21^ | 2.7 × 10^-13^ | 7.9 × 10^-21^ | 0.059 |
