# Supplementary material for: Microbial Community Composition in Municipal Wastewater Treatment Bioreactors Follows a Distance Decay Pattern Primarily Controlled by Environmental Heterogeneity
Source: mSphere. 2021 Oct 20;6(5):e00648-21. doi: 10.1128/mSphere.00648-21 (PMC8527990; doi:10.1128/mSphere.00648-21)
Supplement: FIG S5 [file msphere.00648-21-sf005.docx]

**FIG S5.** Monthly spatial community shift rate (β) when the whole community dissimilarity was calculated with (A) Bray-Curtis (phylotype-based), (B) Sørensen (phylotype-based), and (C) weighted Unifrac indices. Monthly spatial community shift rate (β) of (D) universal colonizers and (E) ubiquitous phylotypes when calculated with Bray-Curtis dissimilarity indices. Distance decay relationships that were not statistically significant was excluded (Pearson’s correlation *P* ≥ 0.001; *P*-values are tabulated in Table S5).
